# P-756. Dalbavancin Use at a Large Academic Health System

**DOI:** 10.1093/ofid/ofaf695.967

**Published:** 2026-01-11

**Authors:** Matthew Donnelly, Yanina Dubrovskaya, Cristian Merchan, Samantha Smalley, Justin Siegfried, Dana Mazo, Kassandra Marsh, Arnold Decano

**Affiliations:** USC / Los Angeles General, Los Angeles, CA; NYU Langone Health, New York, NY; NYU Langone Health, New York, NY; NYULH, New York, New York; NYU Langone Health, New York, NY; New York University, New York, NY; NYU Langone Health, New York, NY; NYU Langone Health, New York, NY

## Abstract

**Background:**

Dalbavancin offers an alternative to standard IV antibiotic therapy with its long half-life. Our objective was to evaluate patients who received dalbavancin (DAL) in the emergency department (ED) for acute bacterial skin/skin structure infection (ABSSSI) and to determine the appropriateness of treatment in compliance with clinical decision support (CDS) criteria. We also evaluated the inpatient use of DAL.

**Table 1:**
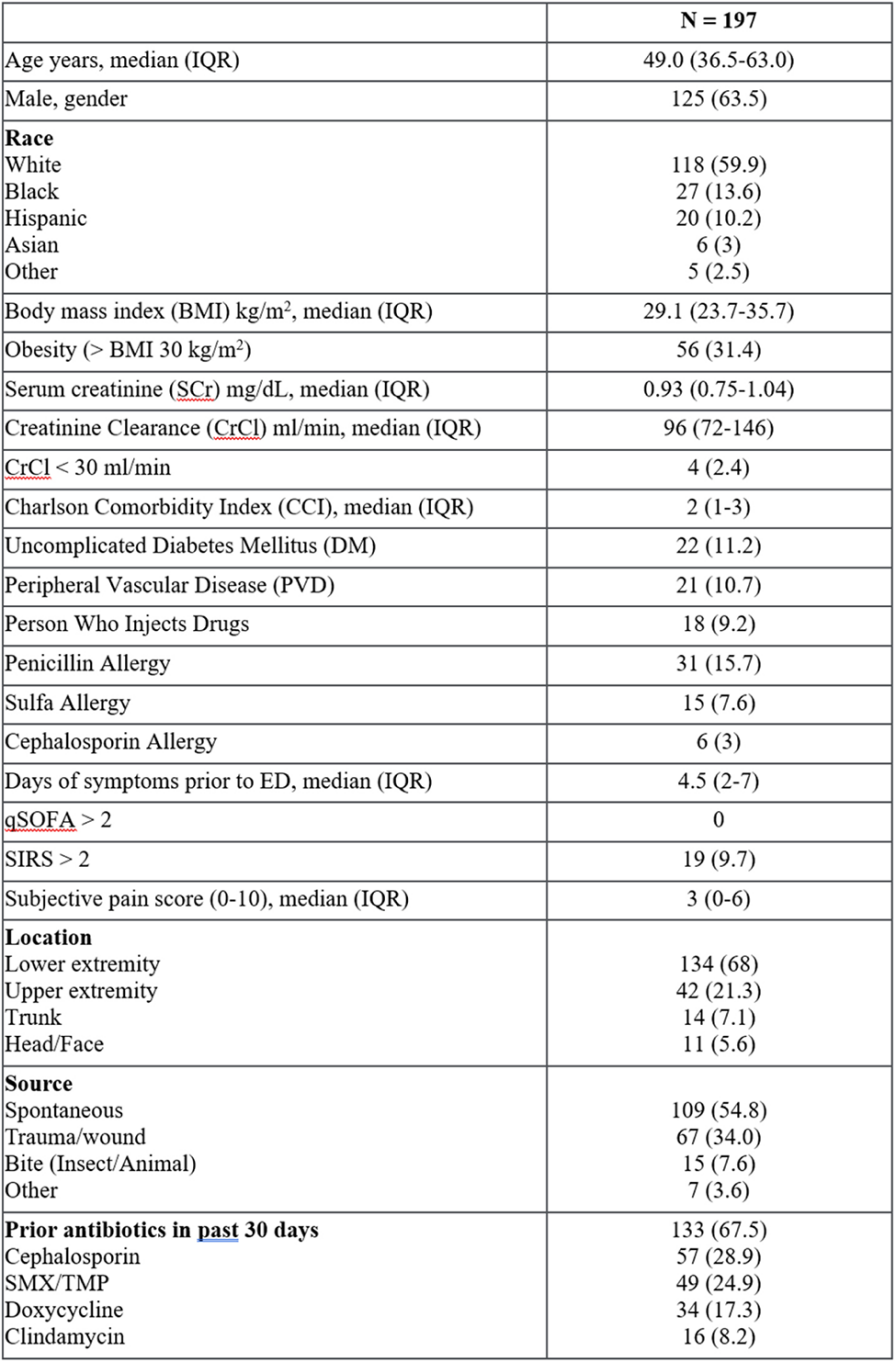
Baseline Characteristics for ED Administrations Variables reported in n (%) unless otherwise stated. qSOFA; quick sepsis related organ failure assessment, SIRS: systemic inflammatory response syndrome

**Table 2:**
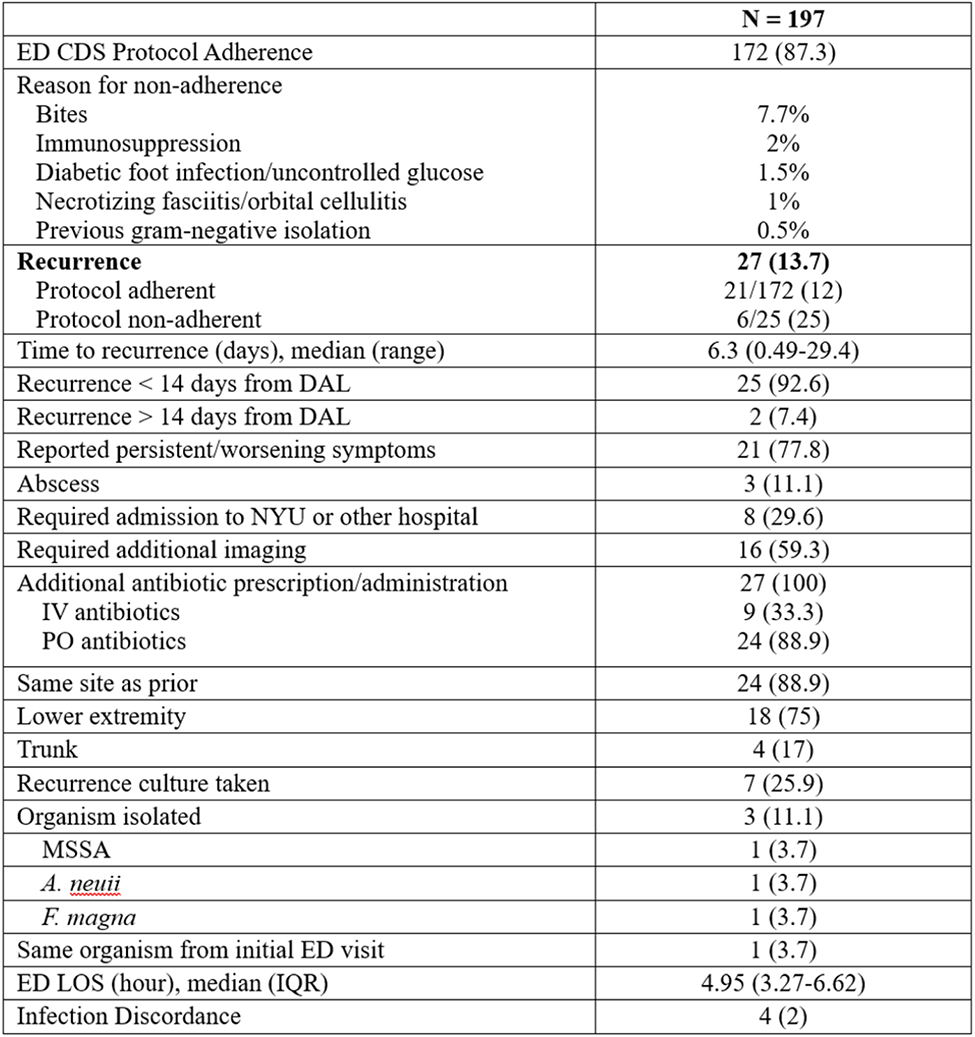
Outcomes for ED Administrations Variables reported in n (%) unless otherwise stated.

**Methods:**

This was a retrospective chart review of patients with documented DAL administration in the ED or an inpatient unit from March 2018 to December 2024. The primary outcome was compliance with DAL CDS use criteria. Secondary outcomes included infection-microbiological discordance, time to recurrence, ED and hospital length of stay (LOS), and alternative use indications.

**Table 3:**
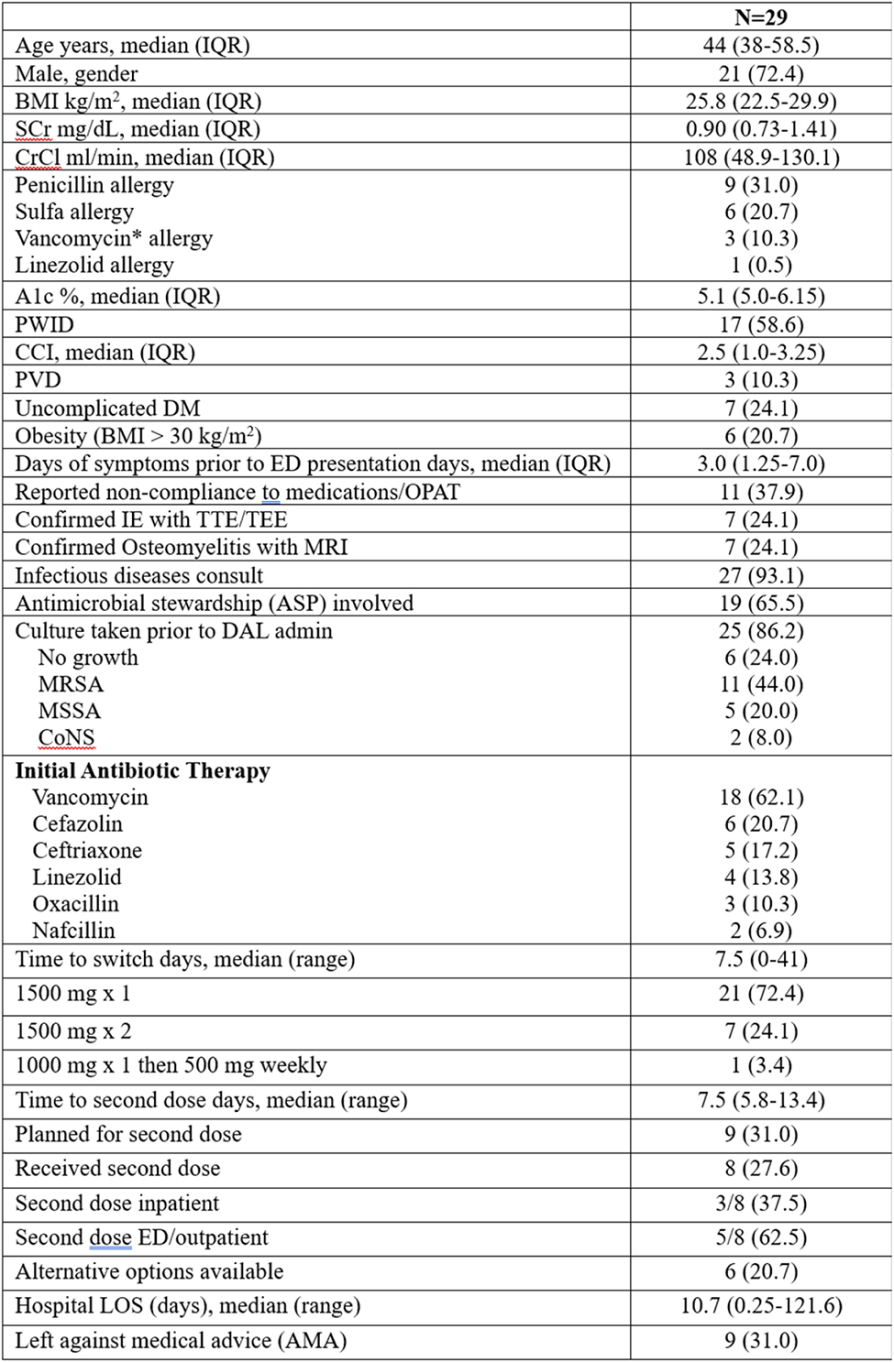
Baseline Characteristics for Inpatient Administrations Variables reported in n (%) unless otherwise stated. *AKI N = 1, Hearing loss/infusion reaction N = 1, Rash/skin peeling N = 1

**Table 4:**
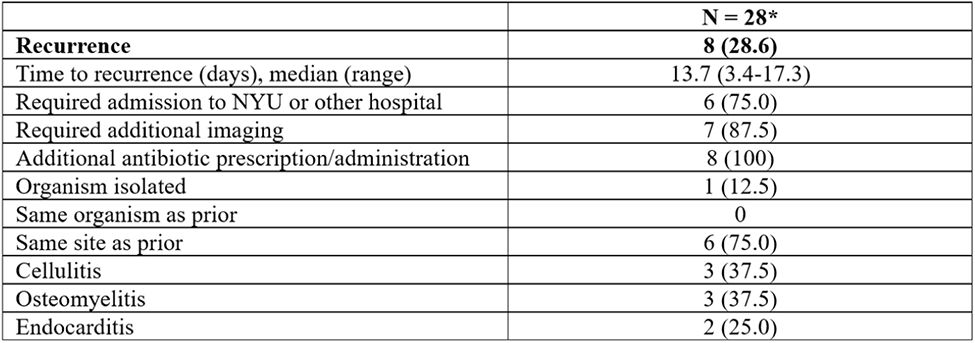
Outcomes for Inpatient Administrations Variables reported in n (%) unless otherwise stated. *One patient was excluded due to administration occurring less than 30 days before collection.

**Results:**

A total of 197 patients were included. Most patients (67.5%) received antibiotics in the past 30 days. Spontaneous (54.8%) and traumatic (34.0%) were the most common etiologies of ABSSSI. The ED CDS protocol adherence rate was 87.3%. The most common reasons for protocol deviation were bite (7.7%), immunosuppression (2%), and diabetic foot infection/uncontrolled glucose (1.5%). Recurrence occurred in 13.7% of patients, with a median time to recurrence of 6.3 days (IQR 2.6-10.3) and occurred more frequently in non-protocol adherent vs. adherent administrations (25% vs. 12%). Twenty-nine patients receiving DAL inpatient were also analyzed. MRSA (44.0%), MSSA (20.0%), and CoNS (8.0%) were the most identified organisms. Seventeen (58.6%) inpatients receiving DAL were people who inject drugs, 11 (37.9%) had reported non-compliance to medications/outpatient antimicrobial therapy (OPAT), and 9 (31.0%) left against medical advice. Most patients were on vancomycin prior to administration (62.1%) with a median time to switch of 7.5 days. Twenty-one (72.4%) patients received one dose of 1500 mg, and 7 (24.1%) received 2 doses of 1500 mg. Infection recurrence occurred in 8 (28.6%) patients.

**Conclusion:**

This study demonstrated a high compliance rate to ED CDS criteria for outpatient dalbavancin use and provides insight on gaps to adherence. Also highlighted is the benefit of inpatient administration in patients with non-compliance or barriers to OPAT/PO therapies for complicated infections.

**Disclosures:**

All Authors: No reported disclosures

